# Ultrasound-guided Microbubble in the Treatment of Cancer: A Mini Narrative Review

**DOI:** 10.7759/cureus.3256

**Published:** 2018-09-04

**Authors:** Minu Ambika Rajendran

**Affiliations:** 1 Biochemistry, California State University, Long Beach, USA

**Keywords:** microbubble, cancer treatment, ultrasound-targeted microbubble, drug delivery

## Abstract

Microbubble is an emerging modality in the field of Medicine for treatment and imaging. Ultrasound-guided microbubble is an effective diagnosing and treatment technique as it can reduce the systemic toxicity of chemotherapeutic drugs. It is also used in targeted gene delivery in gene therapy. The objective of the review article is to formulate a narrative review on the emerging importance of microbubbles in the diagnosis and treatment of cancer and its future in cancer management. The article focuses on the effectiveness of ultrasound-targeted microbubble in the treatment of malignancy.

## Introduction and background

Microbubble is useful for targeted drug delivery [1]. Microbubbles are utilized to transport a drug or gene to a specific area of interest, and then ultrasound is used to break the microbubbles, which leads to the delivery of the bioactive matter [2]. Usage of Ultrasound Exposed Microbubble will increase the efficacy of localized drug delivery and decrease the drug dosage and toxicity of the chemotherapeutic drug [3]. Microbubbles are small, gas-filled bubbles, with a diameter typically between 0.5 µm and 10 µm, that are commonly used as contrast agents in medical imaging and as carriers for targeted drug delivery [4]. Microbubble is a new modality in the treatment of cancer. Microbubble consists of a core filled with gas and an outer covering made of polymers, lipopolymers, lipids, proteins, surfactants or a combination of these [1,4]. Microbubbles were initially developed to enhance ultrasound scans in the 1990s [4]. In recent years, however, studies have shown that ultrasound microbubbles can perform as an important and noninvasive tool in cancer therapy and the use of ultrasound microbubbles to deliver chemotherapeutic drugs to malignant tissues is a promising and evolving prospect [5].

## Review

The diameter of a microbubble, being less than 10 μm approximately, equals the size of a red blood cell and it exhibits a similar rheology in the blood vessels and capillaries in the body [1]. Even though it was initially developed as a method to aid in diagnosing, its use in delivering drugs to targeted tissues is currently more explored. Chemotherapeutic drugs are effective in destroying cancer cells but generate numerous side effects. Microbubbles help in decreasing the side effects of chemotherapy by encapsulating the drug within the shell and releasing the drug on the specific site thereby minimizing the drug contact with the normal tissues [6]. Recent growth in the ultrasound and microbubble-mediated drug delivery technology can improve targeted drug delivery thereby reducing drug dose and adverse effects [7]. This is carried out through a mechanism called sonoporation which is the formation of openings in the blood vessels induced by oscillations triggered by ultrasound and the destruction of microbubbles [7].

A case report published in China described the use of low-frequency ultrasound combined with microbubbles in the treatment of inoperable hepatic cancer [8]. After the treatment, even though the size of the tumor increased minimally, the intensity and enhancement of the tumor were decreased as seen during post-treatment imaging [8]. Furthermore, the abdominal lymph node and the tumor marker level decreased compared to the pre-treatment levels [8].

A clinical case study conducted on patients with pancreatic cancer demonstrated the effectiveness of the combination of ultrasound, microbubbles, and gemcitabine in decreasing the tumor size. Being a very aggressive tumor, it is very rare to see a decrease in the size of the tumor by cancer drugs. Surprisingly, combination therapy using ultrasound and microbubbles showed a decrease in tumor growth. The patients in the treatment group were able to undergo more cycles of treatment and the overall quality of life improved. Furthermore, the patients did not notice any discomforts during the treatment cycle [9].

Many studies utilized the blood vessel destructing properties of microbubbles and ultrasound in the treatment of prostate cancer. One remarkable study explored the effect of low-frequency low-intensity ultrasound with microbubble on the hypoxia environment of prostate cancer [10]. Another noteworthy study analyzed the role of UDP glycosyltransferase 8 (UGT8) in the responses of prostate tumor to ultrasound-stimulated microbubble radiation enhancement therapy [11].

Different studies demonstrated the effectiveness of ultrasound targeted microbubbles in the treatment of ovarian cancer. One experiment demonstrated the effectiveness of paclitaxel-loaded microbubbles coated with Luteinizing hormone releasing hormone analogue and ultrasound-targeted microbubble destruction greatly enhanced the therapeutic effect of paclitaxel [12]. Another study showed that delivering siRNA (small interfacing RNA) with a method using an arginine-grafted bioreducible polymer (ABP), microbubbles (MB), and ultrasound technology (US) together was more effective in ovarian cancer treatment [13].

Ultrasound-targeted microbubble destruction can be used as a noninvasive way in the treatment of breast cancer. A study noted that there was tumor regression by using ultrasound targeted delivery of miR‐133a‐MB without significant side effects [14]. US targeted microbubble is a safe and effective way to transport micro RNAs (miRNA).

Studies have shown that Doxorubicin, one of the widely used antineoplastic drug, can be delivered to the tumor site as Doxorubicin-liposome-containing microbubbles with the use of ultrasound [15]. Ultrasound-mediated microbubble delivery reduced the cytotoxic effect of Doxorubicin in non-cancer cells. In comparison with Doxorubicin-liposomes, DOX-liposome-containing microbubbles destroyed at least two times more melanoma cells after the exposure to ultrasound [15].

Microbubble-mediated ultrasound therapy with Cisplatin or Cetuximab has shown to decrease the tumor size significantly in head and neck squamous cell carcinoma [16].

## Conclusions

Microbubble is a treatment and diagnostic modality that is gaining popularity in the field of cancer treatment. The above-mentioned studies demonstrate the efficacy and scope of microbubbles in cancer detection and treatment. Microbubbles can greatly reduce the side effects of the highly toxic chemotherapeutic drugs as the drug is encapsulated inside the microbubble and will only be released at the desired site. To conclude, ultrasound-guided microbubble is a promising new technique for diagnosing and treating cancer. Further clinical studies and research should be needed to completely know the effectiveness and safety profile of the method. So far, the study results have been encouraging.
